# Lifestyle Behaviours of Children and Adolescents During the First Two Waves of the COVID-19 Pandemic in Switzerland and Their Relation to Well-Being: An Observational Study

**DOI:** 10.3389/ijph.2022.1604978

**Published:** 2022-09-08

**Authors:** Gabriela P. Peralta, Anne-Linda Camerini, Sarah R. Haile, Christian R. Kahlert, Elsa Lorthe, Laura Marciano, Andres Nussbaumer, Thomas Radtke, Agne Ulyte, Milo A. Puhan, Susi Kriemler

**Affiliations:** ^1^ Epidemiology, Biostatistics and Prevention Institute (EBPI), University of Zurich, Zurich, Switzerland; ^2^ Institute of Public Health, Università della Svizzera Italiana, Lugano, Switzerland; ^3^ Department of Infectious Diseases and Hospital Epidemiology, Children’s Hospital of Eastern Switzerland, St. Gallen, Switzerland; ^4^ Department of Infectious Diseases and Hospital Epidemiology, Cantonal Hospital St. Gallen, St. Gallen, Switzerland; ^5^ Unit of Population Epidemiology, Department of Primary Care Medicine, Geneva University Hospitals, Geneva, Switzerland; ^6^ Harvard T. H. Chan School of Public Health and Dana-Farber Cancer Institute, Boston, MA, United States

**Keywords:** COVID-19, physical activity, well-being, children and adolescents, lifestyle, screen time, sleep

## Abstract

**Objectives:** To describe changes in adherence to recommendations for physical activity (PA), screen time (ST), and sleep duration over the first two waves of the pandemic in Switzerland, and to assess the associations of these lifestyle behaviours with life satisfaction and overall health as well-being indicators.

**Methods:** In this observational study, we included 2,534 participants (5–16 years) from four Swiss cantons. Participants, or their parents, completed repeated questionnaires and reported on their (child’s) lifestyle and well-being, between June 2020 and April 2021. We used linear and logistic regression models to assess the associations between lifestyle and well-being.

**Results:** The percentage of children meeting the recommendations for PA and ST decreased from the pre-pandemic period to the first wave, with a slight recovery during the second wave. Participants meeting all three recommendations during the second wave were more likely to report excellent health (OR: 1.65 [95% CI: 1.00–2.76]) and higher life satisfaction (*β*: 0.46 [0.16–0.77]) in early 2021 than participants not meeting any recommendation.

**Conclusion:** We showed a substantial impact of the COVID-19 pandemic on children’s and adolescents’ lifestyle, and a positive association between meeting lifestyle recommendations and well-being.

## Introduction

Lifestyle, including screen time (ST), physical activity (PA), and sleep, is a key predictor of physical and emotional well-being in children and adolescents [1–3]. Thus, understanding how lifestyle behaviours have changed as the COVID-19 pandemic and its related restrictive measures evolved, is of utmost relevance. Like many countries, the Swiss government imposed a lockdown between March and May 2020 [4]. During that period, classroom teaching was interrupted, and then continued with a partial return to class (i.e., alternating face-to-face and online classes) until the start of the summer break. In August 2020, schools reopened as normal, though with continued prevention measures (e.g., mask wearing, class level quarantine following a single positive case) in place. In autumn and early winter 2020, Switzerland experienced a second wave with one of the highest infections rates worldwide [5]. Although classroom teaching in primary and secondary education was still secured, sports clubs, gyms, and other leisure institutions were closed to some extent (i.e., open only for children under age 16 years) or had restrictions in place, such as no mixing of groups, small group sizes and the banning of sports competitions until early 2021 [6]. Following the decrease of infections, in March 2021 most restrictions were lifted and all extracurricular activities such as rehearsals and training for cultural and sports activities were permitted without restrictions for children and adolescents [6].

There is substantial evidence showing that COVID-19 restrictions during the first wave in spring 2020 resulted in a drastic reduction of PA and an increase in ST among children and adolescents, as well as in a delay of sleep onset and offset resulting in a subtle increase of sleep duration [7]. Also, studies conducted during the pandemic reported an association between changes in lifestyle behaviours and well-being. Maintaining adequate levels of PA was associated with better well-being, while higher levels of ST were related to decreased emotional well-being [8–11], which is in line with research from the pre-pandemic period [1, 12]. However, an important limitation of previous research is the fact that most studies evaluated lifestyle changes only during the period when strict lockdown measures were in place. Very few studies extended their assessment beyond June 2020 [10, 13, 14], and only one small (*n* = 19) longitudinal study assessed lifestyle changes up to 2021 [15]. In addition, studies assessing the associations between lifestyle behaviours and well-being before and during the pandemic were mostly based on a single-behaviour approach and assessed time dedicated to each behaviour rather than adherence to (inter-)national recommendations for children and adolescents [16–18]. There is pre-pandemic evidence supporting that meeting recommendations for more than one lifestyle behaviour is more beneficial for children’s and adolescents’ health and development than meeting the recommendations for a single behaviour [19–22]. A recent study using pre-pandemic data reported that a combination of lower ST and higher PA appears to have joint and positive associations with mental well-being in adolescents [23]. However, the study could not account for the contribution of sleep duration to this association.

Therefore, we aimed to describe changes in adherence to international recommendations for PA, ST, and sleep duration over the first two waves of the COVID-19 pandemic (March–May 2020 and October 2020–January 2021) in children and adolescents aged 5–16 years, and to assess the combined associations of these lifestyle behaviours with overall health and life satisfaction in early 2021.

## Methods

### Study Design and Participants

This observational study is part of Corona Immunitas, a research network that investigates the spread and impact of COVID-19 pandemic in Switzerland [24]. We included children and adolescents from four Swiss cantons participating in this network: Ticino (TI), St. Gallen (SG), Graubünden (GR), and Zurich (ZH), which collected data on lifestyle behaviours and well-being at similar time points during the pandemic. These cantons belong to three out of four language regions (German, Italian and Romansch) in Switzerland and comprise 30% of the Swiss population [25]. In TI, SG, and GR, participants were recruited based on a representative sample of children and adolescents drawn from the Swiss Federal Registry. In ZH, the data came from the Ciao Corona study [26], a cohort of school-aged children from 55 schools. Primary schools were randomly selected from the list of all schools in the canton of ZH and matched with the geographically closest secondary school.

Out of a sample of 3,458 eligible children and adolescents, we included 2,534 participants aged 5–16 years with the first assessment completed between June 2020 and January 2021 and follow-up assessments between January and April 2021, and who provided data on lifestyle behaviours and well-being measures (Supplementary Figure S1). In ZH, parents were asked to complete the questionnaires together with their child. In the other three cantons, parents completed the questionnaires for children aged 5–13 years, while adolescents aged 14–16 years completed the questionnaires on their own. See further details on participant recruitment and data collection in the Supplementary Material.

The study was approved by the Ethics Committee of all cantons (TI: 2020-01514; SG/GR: 2020-01247; ZH: 2020-01336). All participants and/or their parents provided informed consent.

### Measures

#### Lifestyle

**FIGURE 1:**
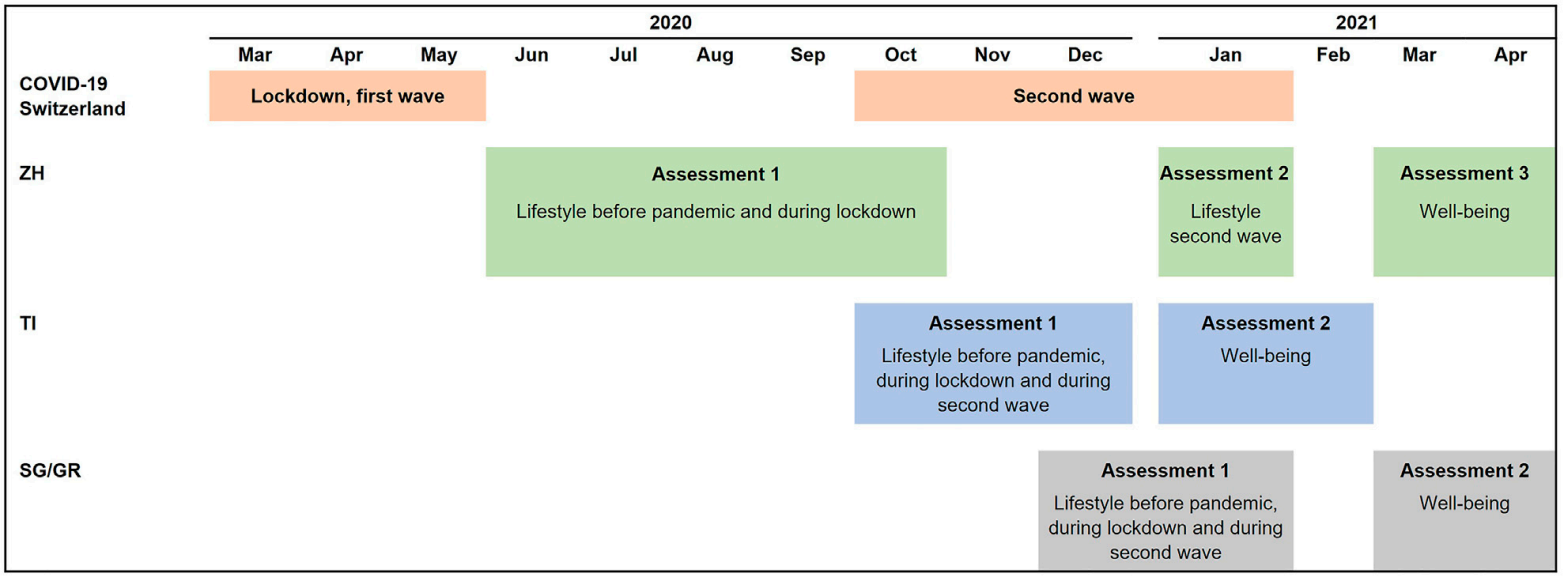
Details on data collection per canton (Corona Immunitas, Switzerland, 2020–2021). Abbreviations: GR, Graubünden: SG, St. Gallen; TI, Ticino; ZH, Zurich. In TI, SG and GR, participants completed questionnaires following a rolling enrolment. Therefore, participants were invited to the second assessment 3 months after the first assessment.

In all cantons, PA, ST, and sleep for the period before the pandemic (i.e., before March 2020) and during the lockdown in the first wave (i.e., between 16 March and 10 May 2020) were assessed retrospectively in the first assessment. Participants from TI, GR and GR reported on their lifestyle for the period during the second wave (i.e., between October 2020 and January 2021) also in the first assessment, while in ZH this was reported in a separate assessment (Figure 1).

PA was assessed by asking how many hours per week, on average, participants spent with PA (at least with light sweating). Weekly hours were converted to daily hours. ST was assessed by asking how many hours, on average, participants spent with electronic media (e.g., smartphone, computer, PlayStation, Xbox, Nintendo, TV) on a typical weekday and on a typical weekend day. Sleep was assessed by asking how many hours, on average, participants slept on a typical weekday and on a typical weekend day. For ST and sleep, we calculated a weighted average as follows: [(weekday*5) + (weekend day*2)]/7. The questions were similar to those used in previous studies assessing lifestyle in children and adolescents [19]. The exact wording of the questions can be found in Supplementary Table S1.

For each time-point, i.e., before the pandemic, during the first wave, and during the second wave, we analysed lifestyle in terms of adherence to international recommendations: ≥1 h/day of PA, ≤2 h/day of ST, and age-specific sleep duration (10–13 h/night for 5 years; 9–11 h/night for 6–13 years; 8–10 h/night for 14–16 years) [16–18]. We also grouped participants into one of the following recommendation adherence patterns: none, PA only, sleep only, ST only, PA and sleep, PA and ST, sleep and ST, and all three.

#### Well-Being

We measured current life satisfaction and overall health as indicators of well-being in a follow-up assessment between January and April 2021. This was the second assessment in TI, SG and GR and the third assessment in ZH. Well-being was thus measured two to 3 months after the assessment of lifestyle behaviours with reference to the second wave (Figure 1).

We assessed life satisfaction with the Cantril ladder [27], a visual scale for rating how children and adolescents perceive their life on a 11-point scale ranging from “0-the worst possible life” to “10-the best possible life.” We assessed overall health by asking “How would you rate your/your child’s health?” Respondents were asked to describe their (child’s) health status on a scale from “1-poor” to “4-excellent.”

Both items have been widely used in epidemiological research [28, 29]. We analysed life satisfaction as a continuous variable and overall health as a binary variable (“excellent” vs. lower ratings).

#### Covariates

We collected participants’ sex, age, height, and weight in the first assessment. We also collected information on parents’ nationality (at least one Swiss; both non-Swiss) and educational level (high: at least one with preparatory high school to university; low/medium: both up to apprenticeship or professional school). We used height and weight to derive age- and sex-specific body mass index (BMI) z-scores by using the World Health Organization child growth standards, and categorised it as underweight, normal weight, and overweight according to the guidelines [30]. We categorised participants’ age according to stages of development: 5–9 years, 10–12 years, and 13–16 years [31]. The three age-groups cover primary school-aged children, tweens, and teens, respectively.

### Statistical Analysis

Changes in lifestyle behaviours during the first and second waves were analysed by descriptive statistics, stratified by age category. We calculated 95% confidence intervals for percentages using the Wilson method [32]. In supplementary analyses, we also stratified changes by sex and analysed lifestyle behaviours as continuous variables.

We analysed the associations of the number of recommendations met and the adherence patterns during the second wave with overall health and life satisfaction in early 2021 using multivariable logistic and linear regression, respectively. We adjusted the models for child’s sex, age category, BMI category, parents’ nationality, parents’ educational level, completion of the questionnaire by parents (i.e., parent report) and canton. To account for previous lifestyle, we also adjusted the models for the number of recommendations met/adherence patterns before the pandemic and during the lockdown of the first wave. We tested interactions between lifestyle behaviours at the second wave and age category. However, the Akaike information criterion and the *p*-value for the likelihood ratio test comparing models with and without interactions indicated that models without interactions fit the data best (data available on request). *p*-values for the adjusted models were interpreted according to levels of significance, as previously recommended [33].

Missing data were reported in the Table 1 and Supplementary Table S2 footnotes. We performed the statistical analysis in R (version 4.0.3) [34] and used *ggplot2* [35] and *patchwork* [36] packages to produce graphs.

**TABLE 1:** Characteristics of the study sample (Corona Immunitas, Switzerland, 2020–2021).

Characteristics	Overall	ZH	TI	SG	GR
	*n* = 2,534	*n* = 1,650	*n* = 640	*n* = 110	*n* = 134
Sex. Girls	1,306 (51.5)	856 (51.9)	313 (48.9)	61 (55.5)	76 (56.7)
Age					
5–9 y	879 (34.7)	539 (32.7)	236 (36.9)	51 (46.4)	53 (39.6)
10–13 y	807 (31.8)	596 (36.1)	146 (22.8)	26 (23.6)	39 (29.1)
14–16 y	848 (33.5)	515 (31.2)	258 (40.3)	33 (30.0)	42 (31.3)
BMI					
Underweight	111 (4.5)	70 (4.3)	35 (5.6)	1 (1.0)	5 (4.0)
Normal weight	1994 (80.0)	1,308 (79.7)	486 (77.6)	88 (88.0)	112 (88.9)
Overweight	389 (15.6)	264 (16.1)	105 (16.8)	11 (11.0)	9 (7.1)
Parental nationality					
Swiss	2,214 (87.6)	1,439 (87.6)	553 (86.4)	100 (90.9)	122 (91.0)
Non-Swiss	312 (12.4)	203 (12.4)	87 (13.6)	10 (9.1)	12 (9.0)
Parental education					
High	1871 (75.3)	1,274 (78.1)	452 (73.5)	69 (63.3)	76 (58.5)
Low/Medium	615 (24.7)	358 (21.9)	163 (26.5)	40 (36.7)	54 (41.5)

Data are n (%). Some percentages do not add up to 100% because of rounding. Some data were missing for BMI (40, 1.6%), parental nationality (8, 0.3%) and parental education (48, 1.9%). There were no missing data for sex and age.

BMI, body mass index; GR, Graubünden; SG, St. Gallen; TI, Ticino; y, years; ZH, Zurich.

## Results

### Description of the Study Sample

We included 2,534 children and adolescents Compared to the study sample, children and adolescents excluded from the analyses were more likely to have both parents of non-Swiss origin and with low/medium education level (Supplementary Table S2). Table 1 shows the main characteristics of the study sample. Most participants had a normal BMI (80%), at least one Swiss parent (87.6%) and at least one parent with high education level (75.3%).

In the follow-up assessment between January and April 2021, 43% of participants reported excellent health. The median life satisfaction score was 8.0 (P_25_–P_75_: 7.0–9.0). Older adolescents (13–16 years) were less likely to report excellent health and had a lower median life satisfaction than younger participants (Table 2).

**TABLE 2:** Description of participants’ well-being measures between January and April 2021 by age group (Corona Immunitas, Switzerland, 2020–2021).

	5–9 y	10–12 y	13–16 y	Total
Excellent health, n (%)	437 (49.8)	357 (44.4)	296 (34.9)	1,090 (43.1)
Life satisfaction,^a^ median (P_25_–P_75_)	8.0 (8.0–9.0)	8.0 (8.0–9.0)	8.0 (7.0–9.0)	8.0 (7.0–9.0)

a Range: 0-worst possible life to 10-best possible life.

*n* = 2,530 for overall health and *n* = 2,528 for life satisfaction.

P_25_, percentile 25th; P_75_, percentile 75th; y, years.

### Changes in PA, ST, Sleep, and Number of Recommendations met

**FIGURE 2:**
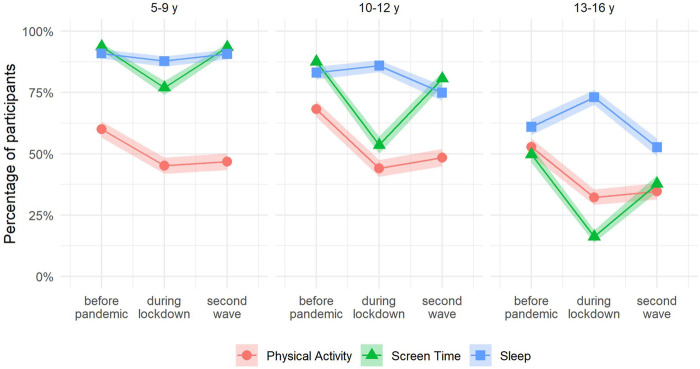
Prevalence of participants meeting recommendations for physical activity, screen time and sleep duration by age group (Corona Immunitas, Switzerland, 2020–2021). Abbreviations: PA, physical activity; sleep, sleep duration; ST, screen time; y, years. Time points: before pandemic: before March 2020; during lockdown: between 16 March and 10 May 2020; second wave: between October 2020 and January 2021. Shaded area represents 95% confidence intervals. We classified participants as meeting recommendations according to international guidelines: ≥1 h/day of PA, ≤2 h/day of ST, and the recommended range by age-groups for sleep duration (i.e., 10–13 h/nigh of sleep for age 5 years, 9–11 h/night for ages 6–13 years, 8–10 h/night for ages 14–16 years).

Across all age groups, we observed a decrease in the percentage of participants meeting the individual recommendations for PA and ST between the pre-pandemic period and the lockdown period (Figure 2; Supplementary Table S3). While these percentages tended to increase during the second wave, they remained below the pre-pandemic levels among the 10- to 12-year-olds and the 13- to 16-year-olds. In the 5- to 9-year-olds, PA levels, too, remained below the pre-pandemic level, but the percentage of children in that age group meeting the recommendations for ST returned to levels observed for the pre-pandemic period. Adherence to recommendations for sleep also changed over time but the pattern was less consistent among age groups. Among the 10- to 12-year-olds and the 13- to 16-year-olds, the percentage of compliant participants increased slightly during the lockdown, but during the second wave it was lower than before the pandemic. For the 5- to 9-year-olds, the percentage of compliant children tended to remain stable.

**FIGURE 3:**
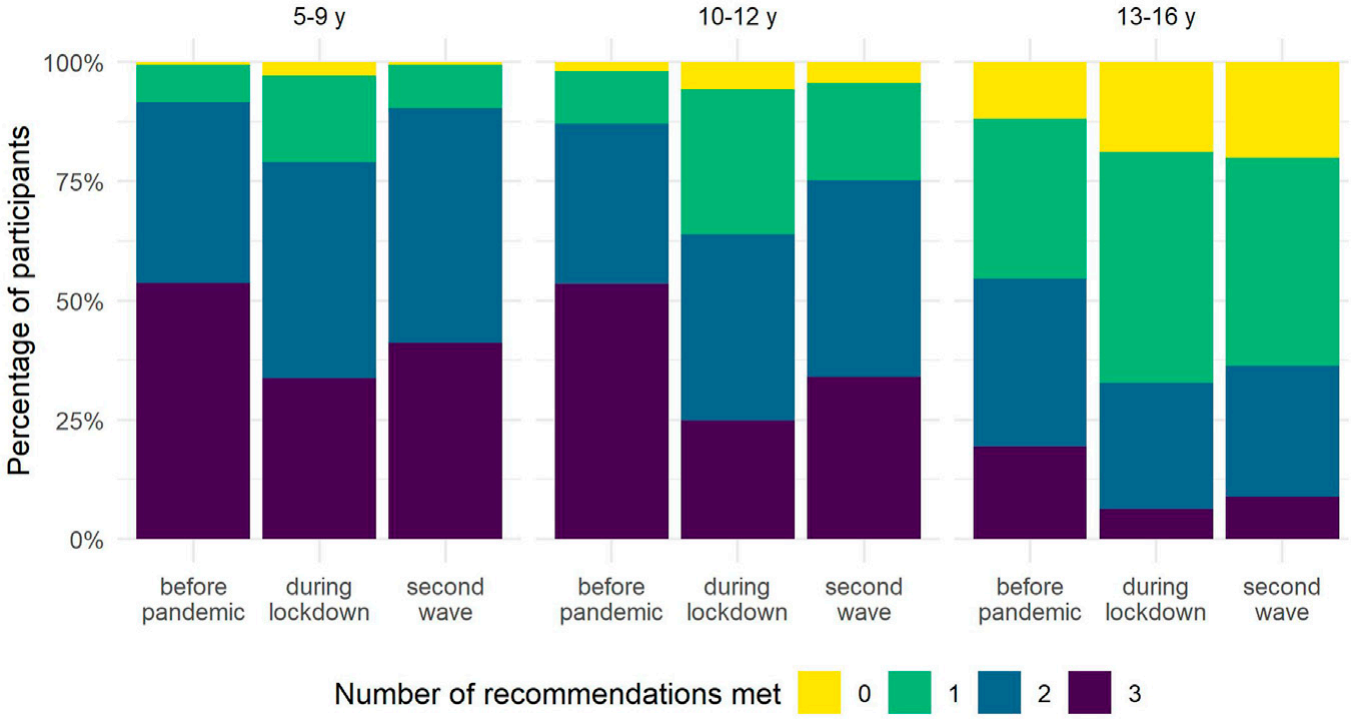
Number of recommendations met by age group (Corona Immunitas, Switzerland, 2020–2021). Abbreviations: y, years. Time points: before pandemic: before March 2020; during lockdown: between 16 March and 10 May 2020; second wave: between October 2020 and January 2021.

The percentage of participants meeting all three recommendations decreased during the lockdown period and increased again during the second wave (Figure 3; Supplementary Table S4). Yet, it remained below the percentages observed for the pre-pandemic period. At all-time points, the 13- to 16-year-olds reported the highest percentage of participants not meeting any of the recommendations.

We observed a similar pattern of change for boys and girls (Supplementary Figures S2, S3). For all age groups and time points, girls were less likely to meet PA recommendations. We observed a similar trend in changes using lifestyle behaviours as continuous variables as described when categorising the variables in terms of adherence to the recommendations (Supplementary Tables S5, S6).

### Associations of Number of Recommendations Met and Adherence Patterns With Well-Being

Table 3 shows the adjusted associations of recommendations met and adherence patterns measured with reference to the second wave with well-being between January and April 2021 (unadjusted associations are presented in Supplementary Table S7). We found that participants meeting recommendations for PA, ST, and sleep during the second wave were almost twice as likely to report excellent health (OR: 1.65, 95% CI: 1.00 to 2.76, *p*-value: 0.054), though the association was weak. Furthermore, those meeting recommendations for all three lifestyle behaviours had, on average, a 0.46 (95% CI: 0.16 to 0.77, *p*-value: 0.003) unit higher life satisfaction score than those not meeting any recommendation. The ORs for excellent health and the regression coefficients for life satisfaction increased relative to the number of recommendations met (Table 3).

**TABLE 3:** Adjusted associations of number of recommendations met and adherence patterns during the second wave of the pandemic with well-being in early 2021 (Corona Immunitas, Switzerland, 2020–2021).

	Excellent health		Life satisfaction	
	aOR (95% CI)	*p*-value	*β* (95% CI)	*p*-value
Number of recommendations met				
No recommendation met	Reference		Reference	
One recommendation met	1.16 (0.75–1.83)	0.508	0.18 (−0.08–0.45)	0.171
Two recommendations met	1.38 (0.86–2.24)	0.184	0.38 (0.10–0.66)	0.009
All three met	1.65 (1.00–2.76)	0.054	0.46 (0.16–0.77)	0.003
Adherence patterns				
No recommendation met	Reference		Reference	
PA only	0.92 (0.48–1.74)	0.788	0.17 (−0.21–0.55)	0.374
Sleep only	1.20 (0.71–2.06)	0.495	0.14 (−0.18–0.46)	0.380
ST only	1.23 (0.73–2.07)	0.443	0.25 (−0.07–0.56)	0.121
PA + Sleep	1.70 (0.94–3.12)	0.083	0.35 (−0.02–0.72)	0.062
PA + ST	1.42 (0.79–2.57)	0.244	0.51 (0.15–0.87)	0.006
Sleep + ST	1.37 (0.82–2.30)	0.237	0.34 (0.03–0.65)	0.031
All three met	1.66 (0.99–2.82)	0.056	0.44 (0.13–0.75)	0.006

Models were adjusted for child’s sex, age category, BMI category, parental nationality, parental educational level, parent report and canton. Models were also adjusted for number of recommendations/adherence patterns met before the pandemic and during the lockdown of the first wave.

PA, physical activity; sleep, sleep duration; ST, screen time; β, regression coefficient; CI, confidence interval; aOR, adjusted odds ratio.

We also observed an association between adherence patterns of lifestyle behaviours during the second wave and well-being in early 2021 (Table 3). In addition to participants meeting the recommendations for all three lifestyle behaviours, participants meeting recommendations for PA and sleep, PA and ST, and sleep and ST had higher average life satisfaction scores than those not meeting any recommendation. We observed a similar pattern for self-reported health, but evidence for an association with the combination of PA and sleep was weak, and there was little evidence for an association with the rest of combinations of lifestyle behaviours.

## Discussion

In this observational study, we found that the first two waves of the COVID-19 pandemic affected lifestyle behaviours in 5- to 16-year-olds in Switzerland. During the lockdown period between March and May 2020, the percentage of children and adolescents meeting the recommendations for PA and ST decreased notably. Although it partially recovered during the second wave of the pandemic between October 2020 and January 2021, the percentage remained below the pre-pandemic period, especially for PA and for those aged 10 years or older. Changes in sleep duration were less marked and they were heterogeneous among age groups. Furthermore, we found an association between the number of recommendations met for lifestyle behaviours during the second wave and well-being assessed between January and April 2021. Participants meeting the recommendations for all three lifestyle behaviours or for combinations of two of them in the second wave had a higher life satisfaction score and were more likely to report excellent health, than those not meeting any recommendation.

Our findings of worsened lifestyle behaviours during the lockdown period are in line with previous cross-sectional and longitudinal studies in children and adolescents [7]. In contrast to previous studies, we did not only analyse early phases of the pandemic but extended our assessment until early 2021. This allowed us to show that, despite the reopening of schools and extracurricular activities for children and adolescents up to 16 years of age, children and adolescents continued to have a more sedentary lifestyle during the second wave of the pandemic compared to the pre-pandemic period, but partially recovered from strict containment measures during the lockdown period. During the second wave, schools were open and classroom teaching was secured for primary and secondary students, which probably played an important role in the reduction of ST during that period. However, since extracurricular activities such as sports training continued to have strict restrictions on place including reduced times and numbers of participants, the options to be physically active were still limited, especially indoors and during the winter months. In addition, due to the high incidence of cases during the second wave, parents may have felt less confident letting their children participate in group or indoor activities. Also, it is possible that the increase in screen-related leisure activities resulted in the reduction of PA at least in part, as evidenced by the negative association between total ST and PA [37]. The shift towards a more sedentary lifestyle during the pandemic, despite some recovery during the second wave, is of concern for children’s and adolescents’ health and development. A cohort study of children in Austria reported that COVID-19 mitigation measures in 2020 were associated with an increase in the proportion of children with overweight and in a reduction of cardiorespiratory fitness [38], which may be partially explained by the reduction of PA and a concomitant increase in sedentary behaviour. Another study in school-aged children in China showed that home confinement during the pandemic was associated with an increase in the prevalence of myopia likely due to significantly decreased time spent outdoors and increased ST [39]. It is also worrying that the pandemic exacerbated the sedentary lifestyle of older adolescents. Only a small percentage of adolescents aged 13–16 years, met recommendations for PA and ST before the pandemic, and even a lower percentage met recommendations for all three lifestyle behaviours. Restrictions during the lockdown period resulted in only 32% and 16% of older adolescents complying with PA and ST recommendations, respectively. The shift towards an even more sedentary lifestyle during the pandemic may place adolescents at an increased risk for deleterious long-term health effects such as cardio-metabolic risk and mental health problems.

We found that the pandemic also led to a change in adherence to recommendations for sleep in adolescents, but not in children aged 5–9 years. During the lockdown, the percentage of adolescents aged 10–12 and 13–16 years meeting the recommendations for sleep was slightly higher than before the pandemic, consistent with previous literature [40]. In contrast, during the second wave, the percentage of compliant adolescents was lower than in the pre-pandemic period. This reduction may be explained by the increase in ST. Previous research reported that adolescents who spent more time on screens slept fewer hours, primarily due to time spent on portable devices such as phones, which proved to delay sleep onset [41]. During the lockdown period, the irregular timetable and the absence of travel to school, may have allowed the delay in sleep time to be compensated by a later waking time, resulting in an overall increase in sleep duration. However, it is likely that this compensation may not have been possible during the second wave due to the return to a more stable schedule while schools were kept open.

In our population, lifestyle was associated with two indicators of well-being, self-rated health and life satisfaction, which is consistent with previous research conducted before [1, 12, 23] and during the pandemic [8–10]. In contrast to previous studies, we accounted for the combined associations of PA, ST, and sleep duration with well-being, and demonstrated that meeting recommendations for all three behaviours as well as for combinations of two of them is more relevant for achieving optimal well-being than meeting recommendations for just a single behaviour. Evidence for an association with number of recommendations met and adherence patterns was stronger for life satisfaction than for self-reported health. Our findings support previous literature showing a dose-response relationship between lifestyle behaviours and physical health or global cognition [19, 20]. Importantly, lifestyle behaviours during the second wave were associated with well-being in early 2021, even after accounting for lifestyle before the pandemic and during the lockdown of the first wave. This suggests that well-being in children and adolescents is associated with recent lifestyle behaviours and thus likely fluctuating as these behaviours change. To note, well-being was assessed in a separate follow-up questionnaire two to 3 months after the assessment of lifestyle behaviours during the second wave, thus ruling out potential bias attributable to cross-sectional designs. The fact that schools were open during the second wave of the pandemic as well as at the time when well-being was assessed (i.e., early 2021) may have played an important role in the observed associations. Schools, as well as leisure facilities, not only have an important role in promoting healthy lifestyle behaviours but are also essential to promote children’s and adolescents’ mental health and well-being [42].

### Future Directions for Practice and Research

Given the already high prevalence of children and adolescents not meeting lifestyle recommendations in the pre-pandemic period and the worsening during the pandemic in 2020, public health policies aiming to avoid permanent changes in lifestyle and negative long-term consequences associated with adverse changes during the pandemic are needed. Our findings suggest that the drastic changes in lifestyle behaviour during the early pandemic may have been partially recovered with the lifting of pandemic related restrictions. However, variability in recovery may be large and put those not meeting lifestyle recommendations before the pandemic at even higher risk for health problems. Future research should test intervention strategies to revert lifestyle changes using specific approaches for different age groups, especially for adolescents and children with high-risk behaviours (i.e., insufficient PA and/or sleep, and high media use). In addition, it is important that future studies make a special effort to include families from low socioeconomic status and collect information on socioeconomic factors that may influence the association between lifestyle behaviours and well-being such as parental working conditions or availability of green spaces nearby. Children and adolescents with a low socioeconomic status are likely to have a more sedentary lifestyle and more mental health problems than those from affluent families [43, 44]. Therefore, it is possible that these children and adolescents are more affected by restrictions in their daily life activities. Finally, future research should assess the long-term consequences of the pandemic on lifestyle behaviours and extend the assessment to the period when all restrictions are lifted and, eventually, after the pandemic.

### Strengths and Limitations

The strengths of this study are the large sample size and the broad age range of the study sample, which allowed us to assess changes in lifestyle behaviours separately for different age groups. The inclusion of children and adolescents from four geographically and culturally different cantons of Switzerland support the external validity of our results, although we recognise that this is not a truly population-based study. In addition, the availability of measurements of lifestyle behaviours with reference to three time-points (i.e., pre-pandemic, lockdown, and second wave) allowed us to characterize changes over time and the partial recovery with the relief of pandemic related restrictions. Finally, as we had data for PA, ST and sleep, we were able to assess the associations between these lifestyle behaviours alone and in combination with well-being measured in early 2021.

However, we also need to acknowledge some limitations. Lifestyle behaviours were self-reported or based on parental-report. Also, these factors were assessed retrospectively for the period before the pandemic and during the lockdown of the first wave and the length of recall was different for each canton. Therefore, there is a potential bias in the estimation of PA, ST, and sleep. The slight differences in the formulation of the questions to assess lifestyle behaviours is another limitation. In ZH, questions for PA referred to hours per day (instead of hours per week), which could have led to an overestimation of PA for this subgroup. In addition, we did not collect data on frequency of PA or type of ST, although it has been suggested that both of these factors play a role on children’s and adolescents’ mental health [45, 46]. Furthermore, well-being indicators were also reported by parents for younger children and therefore some bias is possible for these measures, though we adjusted models for parent report. The fact that participants included in the study were more often from families with Swiss nationality and a high education level than those excluded due to missing data may not allow the generalizability of our results to populations with more ethnic variability and lower socio-economic status. Finally, although we accounted for a wide range of potential confounders when assessing the associations between lifestyle and well-being, residual confounding may still be a concern as we did not consider other factors such as COVID-19 infections, existing psychological problems, social isolation, parental working conditions, mental health or family cohesion, some of which were likely to change over the course of the pandemic Future studies should repeatedly assess and model covariates that are subject to change to evaluate their influence on the association between lifestyle and well-being in children and adolescents.

### Conclusion

In conclusion, our findings demonstrate that the COVID-19 pandemic has had a negative effect on children’s and adolescents’ lifestyle behaviours, but some recovery has taken place within the first year since the outbreak. Policymakers and school managers should tease out the balance of disease prevention and promotion of a healthy lifestyle when (re-)activating restrictive measures. In addition, our study indicates that lifestyle is an important predictor of children’s and adolescents’ well-being, and it further suggests that future public health strategies aiming to promote well-being should target sufficient time for PA and sleep as well as reduce ST.
