# Supplementary material for: Lifestyle Behaviours of Children and Adolescents During the First Two Waves of the COVID-19 Pandemic in Switzerland and Their Relation to Well-Being: An Observational Study
Source: Int J Public Health. 2022 Sep 8;67:1604978. doi: 10.3389/ijph.2022.1604978 (PMC9496876; doi:10.3389/ijph.2022.1604978)
Supplement: Supplementary file 1 [file DataSheet1.docx]

**SUPPLEMENTARY FILE:**

**Lifestyle behaviours of children and adolescents during the first two waves of the COVID-19 pandemic in Switzerland and their relation to well-being: an observational study**

**Supplementary file to publication:**

Peralta GP, Camerini A-L, Haile SR, Kahlert CR, Lorthe E, Marciano L, Nussbaumer A, Radtke T, Ulyte A, Puhan MA and Kriemler S (2022) Lifestyle Behaviours of Children and Adolescents During the First Two Waves of the COVID-19 Pandemic in Switzerland and Their Relation to Well-Being: An Observational Study. *Int J Public Health* 67:1604978. doi: 10.3389/ijph.2022.1604978

**Table of contents**

[Description of study participants and data collection per canton 3](#_Toc112083245)

[Table S1. Question formulation for lifestyle behaviours per canton (*Corona Immunitas*, Switzerland, 2020-2021) 5](#_Toc112083246)

[Figure S1. Flowchart of the study population (*Corona Immunitas,* Switzerland, 2020-2021) 6](#_Toc112083247)

[Table S2. Characteristics of children and adolescent included and excluded from the analysis (*Corona Immunitas,* Switzerland, 2020-2021) 7](#_Toc112083248)

[Table S3. Prevalence of participants meeting recommendations for physical activity, screen time and sleep duration by age group (*Corona Immunitas,* Switzerland, 2020-2021) 8](#_Toc112083249)

[Table S4. Number of recommendations met by age group (*Corona Immunitas,* Switzerland, 2020-2021) 9](#_Toc112083250)

[Figure S2. Prevalence of participants meeting recommendations for physical activity, screen time and sleep duration by age group and sex (*Corona Immunitas,* Switzerland, 2020-2021) 10](#_Toc112083251)

[Figure S3. Number of recommendations met period by age group and sex (*Corona Immunitas,* Switzerland, 2020-2021) 11](#_Toc112083252)

[Table S5. Levels of physical activity, screen time and sleep duration by age group (*Corona Immunitas,* Switzerland, 2020-2021) 12](#_Toc112083253)

[Table S6. Levels of physical activity, screen time and sleep duration by age group and sex (*Corona Immunitas,* Switzerland, 2020-2021) 13](#_Toc112083254)

[Table S7. Unadjusted associations of number of recommendations met and adherence patterns during the second wave of the pandemic with well-being in early 2021 (*Corona Immunitas,* Switzerland, 2020-2021) 14](#_Toc112083255)

[References 15](#_Toc112083256)

## Description of study participants and data collection per canton

This observational study is part of *Corona Immunitas*, a research network that investigates the spread and impact of COVID-19 pandemic in Switzerland [1]. We included children and adolescents from four Swiss cantons: Ticino (TI), St. Gallen (SG), Graubünden (GR), and Zurich (ZH). These cantons belong to three out of four language regions (German, Italian and Romansch) in Switzerland and include 30% of the Swiss population [2]. Details on participants’ recruitment in each canton are presented below:

TI:

The data for Italian-speaking Switzerland stem from the *Corona Immunitas Ticino* population-based, prospective cohort study. It combines repeatedly collected digital survey and serology data from a representative sample during the course of the pandemic, with a specific focus on children and adolescents between 5 and 19 years of age. A representative sample of 4264 children and adolescents was drawn from the Swiss Federal Registry in May 2020 and invited to the study between September and November 2020. The study excluded people with insufficient Italian skills, under legal guardianship, in an asylum procedure, or with a short-term residency permit (i.e., < 1 year). Of all invited people, 1262 (30%) agreed to participate. Of these, 640 participants aged 5 to 16 years filled out baseline and follow-up questionnaires and provided data on the key variables for the present study. For children aged 5 to 13, parents provided answers with reference to the invited child. For adolescents aged 14 to 16, survey responses were based on self-report.

The study was approved by the Ethics Committee of the Canton of Ticino, Switzerland (2020-1514).

SG and GR:

The data for Eastern-Switzerland was collected over the course of the *Corona Immunitas Ostschweiz* in the cantons of St. Gallen and Graubünden. The study contains a onetime serology and digital survey data with monthly and weekly digital follow-up questionnaires. Based on a randomized sample drawn from the Swiss Federal Registry in October 2020, a total of 3335 children and adolescents aged 5 to 19 years were invited to participate between December 2020 and April 2021. Of all invited children, 523 (16%) agreed to participated. Of these, 244 participants aged 5 to 16 years filled out baseline and follow-up questionnaires and provided data on the key variables for the present study. For children aged 5 to 13, parents provided answers with reference to the invited child. For adolescents aged 14 to 16, survey responses were based on self-report.

The study was approved by the Ethics Committee of the Cantons of St. Gallen and Graubünden, Switzerland (2020-01247).

ZH:

The data for the Zurich canton come from the *Ciao Corona* study, which has been described elsewhere.[3] Briefly, *Ciao Corona* is a longitudinal, school-based observational study in a regionally representative cohort of children and adolescents (aged 6 to 16 years) attending school in the canton of Zurich, Switzerland. The study combines repeatedly collected serological and survey data. Primary schools were randomly selected from the list of all schools in the canton of Zurich, stratified by region, and matched with the geographically closest secondary school. Main exclusion criteria for schools were small school size (<40 children per school level), and for participants—suspected or confirmed infection with SARS-CoV-2 during testing. Of 156 schools invited to participate, 55 schools agreed and 2585 children and adolescents were enrolled from June 16 to July 9, 2020. Of these, 1650 participants filled out baseline and follow-up questionnaires and provided data on the key variables for the present study. Parents/legal guardians of the participating children were invited to fill baseline and follow-up questionnaires online, together with their child.

The study was approved by the Ethics Committee of the Canton of Zurich, Switzerland (2020-01336).

## Table S1. Question formulation for lifestyle behaviours per canton (*Corona Immunitas*, Switzerland, 2020-2021)

Abbreviations: GR: Graubünden: SG: St, Gallen; TI: Ticino; ZH: Zurich

* When asked for weekdays and weekend days separately, we calculated a weighted average as follows: [(weekday*5) + (weekend day*2)]/7.

| **Lifestyle behaviour*** | **ZH** | **TI** | **SG/GR** |
| --- | --- | --- | --- |
| Physical activity | How many hours of sport or other physical activity per day (that caused sweating or breathing more) does your child do on a typical weekday (also counting school sport)?  How many hours of sport or other physical activity per day (that caused sweating or breathing more) per day does your child do on a typical weekend day (also counting school sport)? | On average, during a week, how much time does your child spend with physical activity (at least with light sweating)? | On average, how much time does your child spend being physically active (with at least light sweating) during the week? (Including physical education at school) |
| Sleep | How many hours per day does your child sleep on a typical weekday? | On average, during a normal weekday (Mon-Fri), how many hours does your child sleep?  On average, during a normal weekend day (e.g., Sunday), how many hours does your child sleep? | On average, how many hours does your child sleep on a normal weekday (Mon-Fri)?  On average, how many hours does your child sleep on a normal holiday (e.g., Sunday)? |
| Screen time | How many hours a day does your child use electronic devices (e.g., mobile phone, play-station, Xbox, Nintendo, computer, TV)? | On average, during a normal weekday (Mon-Fri), how many hours does your child spend with electronic media (e.g., smartphone, computer, PlayStation, Xbox, Nintendo, TV?  On average, during a normal weekend day (e.g., Sunday), how many hours does your child spend with electronic media (e.g., smartphone, computer, PlayStation, Xbox, Nintendo, TV? | On average, how many hours does your child use electronic devices (e.g., smartphone, computer, Playstation, Xbox, Nintendo, TV) on a normal weekday (Mon-Fri)?  On average, how many hours does your child use electronic devices (e.g., smartphone, computer, Playstation, Xbox, Nintendo, TV) on a normal holiday (e.g., Sunday)? |


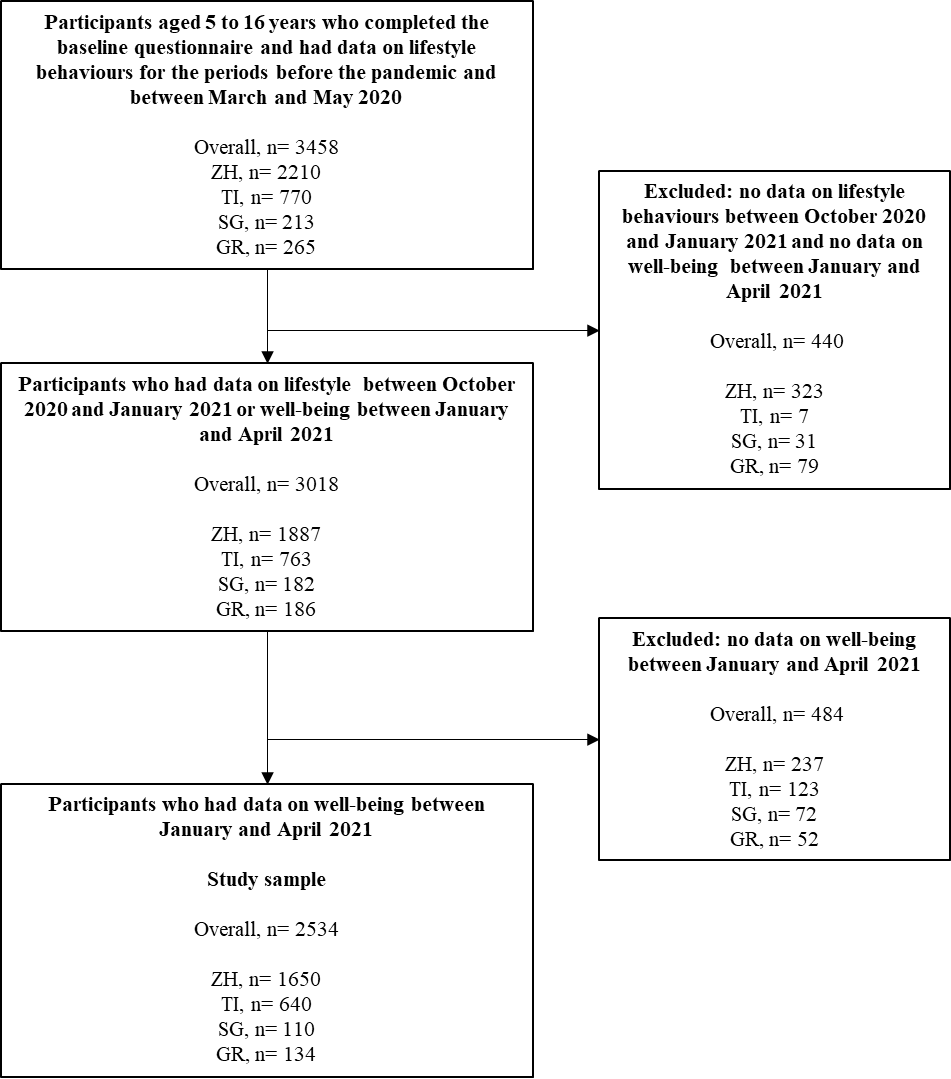


## Figure S1. Flowchart of the study population (*Corona Immunitas,* Switzerland, 2020-2021)

Abbreviations: GR: Graubünden: SG: St. Gallen; TI: Ticino; ZH: Zurich

## Table S2. Characteristics of children and adolescent included and excluded from the analysis (*Corona Immunitas,* Switzerland, 2020-2021)

| **Characteristics** | **Included**  n= 2534 | **Excluded** n= 924 | **p-value** |
| --- | --- | --- | --- |
| **Sex. Girls** | 1306 (51.5) | 459 (49.7) | 0.600 |
| **Age** |  |  |  |
| 5-9 y | 879 (34.7) | 240 (26.0) | <0.001 |
| 10-12 y | 807 (31.8) | 287 (31.1) |  |
| 13-16 y | 848 (33.5) | 397 (43.0) |  |
| **BMI category** |  |  | 0.090 |
| Underweight | 111 (4.5) | 41 (4.5) |  |
| Normal weight | 1994 (80.0) | 697 (76.8) |  |
| Overweight | 389 (15.6) | 170 (18.7) |  |
| **Parental nationality** |  |  | <0.001 |
| Swiss | 2214 (87.6) | 690 (75.2) |  |
| Non-Swiss | 312 (12.4) | 228 (24.8) |  |
| **Parental education** |  |  | <0.001 |
| High | 1871 (75.3) | 580 (65.5) |  |
| Low/Medium | 615 (24.7) | 306 (34.5) |  |

Abbreviations: BMI: body mass index; y: years.

Data are n (%). Some percentages do not add up to 100% because of rounding.

Some data were missing for the participants included (40 for BMI, 8 for parental nationality, and 48 for parental education) and excluded (16 for BMI, 6 for parental nationality, and 38 for parental education) from the analysis.

## Table S3. Prevalence of participants meeting recommendations for physical activity, screen time and sleep duration by age group (*Corona Immunitas,* Switzerland, 2020-2021)

|  | **Age group** | **Before pandemic (0)** | **During lockdown (1)** | **Second wave (2)** | **p-value* 0 *vs.* 1** | **p-value* 0 *vs.* 2** | **p-value* 1 *vs*. 2** |
| --- | --- | --- | --- | --- | --- | --- | --- |
| **PA** | **5-9 y** | 511/852 (60.0%) | 392/868 (45.2%) | 378/808 (46.8%) | <0.001 | <0.001 | 0.310 |
|  | **10-12 y** | 530/776 (68.3%) | 351/797 (44.0%) | 354/731 (48.4%) | <0.001 | <0.001 | 0.027 |
|  | **13-16 y** | 418/792 (52.8%) | 268/832 (32.2%) | 269/777 (34.6%) | <0.001 | <0.001 | 0.070 |
| **ST** | **5-9 y** | 815/869 (93.8%) | 669/869 (77.0%) | 754/806 (93.5%) | <0.001 | 0.660 | <0.001 |
|  | **10-12 y** | 700/799 (87.6%) | 428/799 (53.6%) | 594/736 (80.7%) | <0.001 | <0.001 | <0.001 |
|  | **13-16 y** | 417/838 (49.8%) | 131/811 (16.2%) | 293/777 (37.7%) | <0.001 | <0.001 | <0.001 |
| **Sleep** | **5-9 y** | 795/874 (91.0%) | 767/873 (87.9%) | 737/812 (90.8%) | <0.001 | 0.090 | 0.080 |
|  | **10-12 y** | 660/794 (83.1%) | 681/792 (86.0%) | 552/737 (74.9%) | 0.040 | <0.001 | <0.001 |
|  | **13-16 y** | 512/839 (61.0%) | 611/836 (73.1%) | 412/782 (52.7%) | <0.001 | <0.001 | <0.001 |

Abbreviations: PA: physical activity; sleep: sleep duration; ST: screen time; y: years.

Time points: before pandemic: before March 2020; during lockdown: between 16 March and 10 May 2020; second wave: between October 2020 and January 2021.

Data are n/N (%). *Time point comparisons were tested using the McNemar's test for paired measures.

We classified participants as meeting recommendations according to international guidelines: ≥ 1h/day of PA; ≤2h/day of ST; 10-13 h/nigh of sleep for age 5 years, 9-11 h/night for ages 6-13 years, 8-10 h/night for ages 14-16.

## Table S4. Number of recommendations met by age group (*Corona Immunitas,* Switzerland, 2020-2021)

| **Age group** | **Time point** | **None** | **One** | **Two** | **All three** |
| --- | --- | --- | --- | --- | --- |
| **5-9 y** | **Before pandemic** | 4 (0.5%) | 67 (8.0%) | 319 (38.0%) | 450 (53.6%) |
|  | **During lockdown** | 24 (2.8%) | 155 (18.1%) | 388 (45.4%) | 288 (33.7%) |
|  | **Second wave** | 4 (0.5%) | 73 (9.1%) | 394 (49.3%) | 328 (41.1%) |
| **10-12 y** | **Before pandemic** | 14 (1.8%) | 84 (11.1%) | 255 (33.6%) | 405 (53.4%) |
|  | **During lockdown** | 44 (5.7%) | 236 (30.4%) | 303 (39.0%) | 193 (24.9%) |
|  | **Second wave** | 31 (4.3%) | 149 (20.6%) | 298 (41.2%) | 246 (34.0%) |
| **13-16 y** | **Before pandemic** | 92 (11.8%) | 261 (33.6%) | 274 (35.3%) | 150 (19.3%) |
|  | **During lockdown** | 148 (18.7%) | 384 (48.6%) | 209 (26.5%) | 49 (6.2%) |
|  | **Second wave** | 153 (20.0%) | 335 (43.8%) | 209 (27.3%) | 68 (8.9%) |

Abbreviations: y: years.

Time points: before pandemic: before March 2020; during lockdown: between 16 March and 10 May 2020; second wave: between October 2020 and January 2021.

Data are n (%).

## Figure S2. Prevalence of participants meeting recommendations for physical activity, screen time and sleep duration by age group and sex (*Corona Immunitas,* Switzerland, 2020-2021)

**
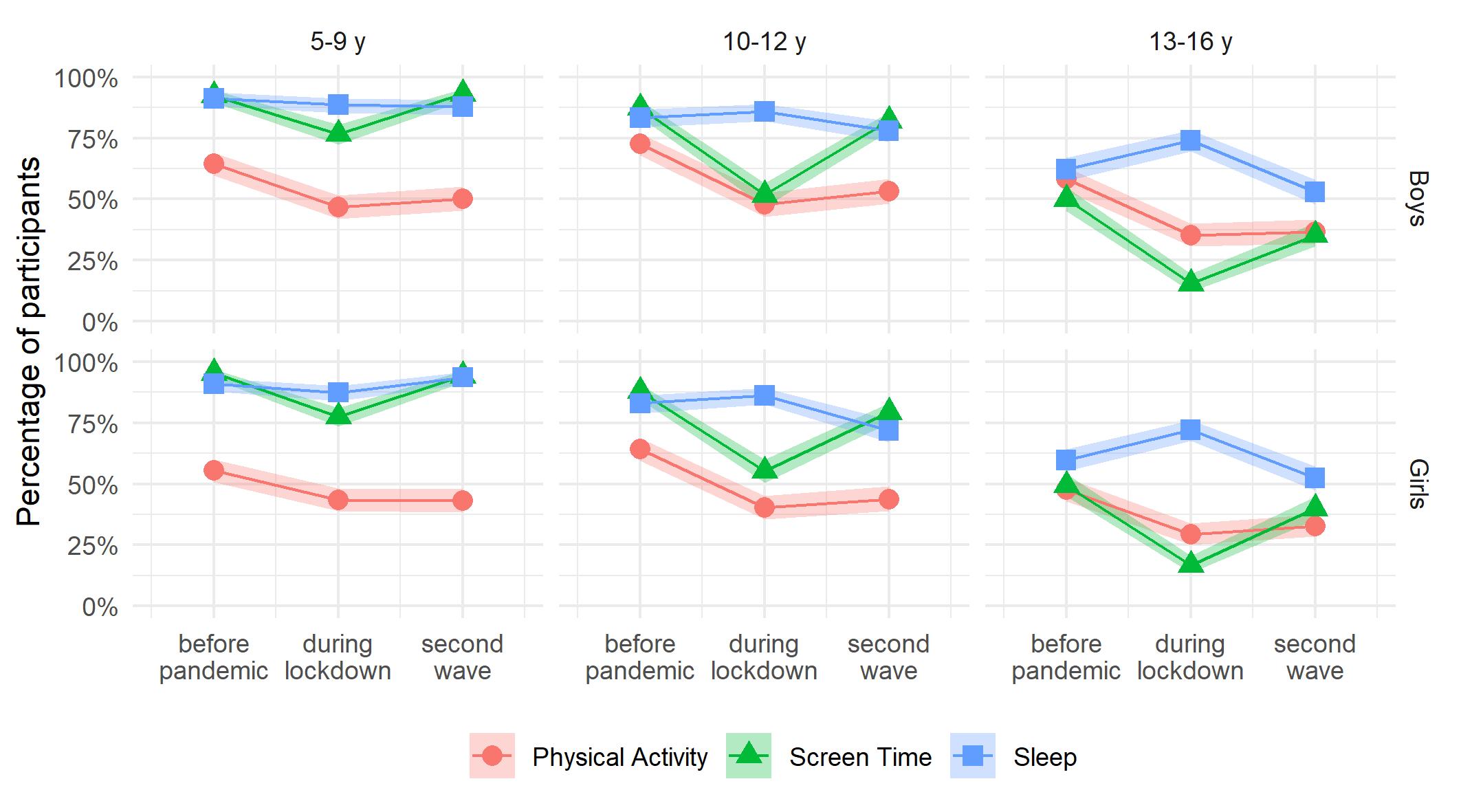
**

Abbreviations: PA: physical activity; sleep: sleep duration; ST: screen time.

Time points: before pandemic: before March 2020; during lockdown: between 16 March and 10 May 2020; second wave: between October 2020 and January 2021.

Shaded area represents 95% confidence intervals. We classified participants as meeting recommendations according to international guidelines: ≥ 1h/day of PA, ≤2h/day of ST, and the recommended range by age-groups for sleep duration (10-13 h/nigh for age 5 years, 9-11 h/night for ages 6-13 years, 8-10 h/night for ages 14-16).

## Figure S3. Number of recommendations met period by age group and sex (*Corona Immunitas,* Switzerland, 2020-2021)

**
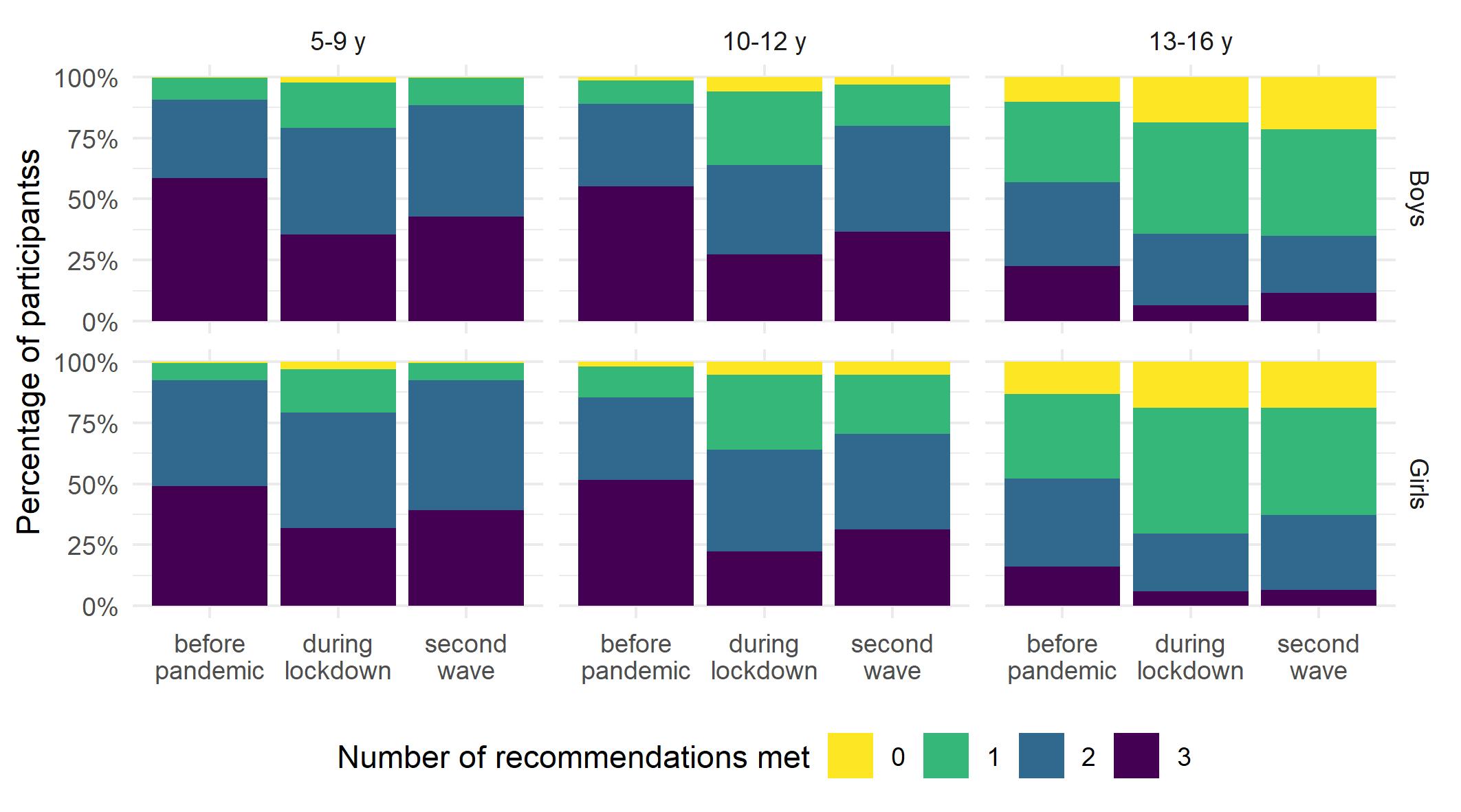
**

Abbreviations: y: years.

Time points: before pandemic: before March 2020; during lockdown: between 16 March and 10 May 2020; second wave: between October 2020 and January 2021.

## Table S5. Levels of physical activity, screen time and sleep duration by age group (*Corona Immunitas,* Switzerland, 2020-2021)

|  |  | **5-9 y, n = 879**  Median (P_25_-P_75_) | **10-12 y, n = 807**  Median (P_25_-P_75_) | **13-16 y, n = 848**  Median (P_25_-P_75_) |
| --- | --- | --- | --- | --- |
| **PA (h/day)** | **Before pandemic** | 1.0 (0.6 - 1.5) | 1.0 (0.8-2.0) | 1.0 (0.6-1.6) |
|  | **During lockdown** | 0.6 (0.3-1.0) | 0.5 (0.3-1.0) | 0.5 (0.2-1.0) |
|  | **Second wave** | 0.9 (0.6-1.3) | 0.9 (0.6-1.4) | 0.7 (0.4-1.3) |
| **ST (h/day)** | **Before pandemic** | 0.6 (0.5-1.0) | 1.0 (0.5-2.0) | 2.3 (1.5-3.3) |
|  | **During lockdown** | 1.1 (0.8-2.0) | 2.0 (1.0-3.6) | 4.5 (3.0-6.0) |
|  | **Second wave** | 0.8 (0.5-1.1) | 1.3 (1.0-2.0) | 3.0 (2.0-4.0) |
| **Sleep (h/night)** | **Before pandemic** | 10.0 (9.5-10.5) | 9.5 (9.0-10.0) | 8.0 (8.0-9.0) |
|  | **During lockdown** | 10.0 (9.5-11.0) | 10.0 (9.0-10.0) | 9.0 (8.0-10.0) |
|  | **Second wave** | 10.0 (9.3-10.3) | 9.0 (9.0-10.0) | 8.0 (7.6-8.9) |

Abbreviations: PA: physical activity; sleep: sleep duration; ST: screen time, h: hours; y: years.

Time points: before pandemic: before March 2020; during lockdown: between 16 March and 10 May 2020; second wave: between October 2020 and January 2021.

## Table S6. Levels of physical activity, screen time and sleep duration by age group and sex (*Corona Immunitas,* Switzerland, 2020-2021)

|  |  | **Girls** | | | **Boys** | | |
| --- | --- | --- | --- | --- | --- | --- | --- |
|  |  | **5-9 y, n = 454** | **10-12 y, n = 411** | **13-16 y, n = 441** | **5-9 y, n = 423** | **10-12 y, n = 396** | **13-16 y, n = 406** |
| **PA (h/day)** | **Before pandemic** | 1.0 (0.6 - 1.5) | 1.0 (0.7 - 1.7) | 0.9 (0.5 - 1.5) | 1.0 (0.7 - 1.8) | 1.3 (0.9 - 2.0) | 1.0 (0.6 - 1.9) |
|  | **During lockdown** | 0.6 (0.3 - 1.0) | 0.5 (0.3 - 1.0) | 0.5 (0.2 - 1.0) | 0.7 (0.3 - 1.4) | 0.7 (0.3 - 1.0) | 0.5 (0.1 - 1.0) |
|  | **Second wave** | 0.9 (0.6 - 1.3) | 0.9 (0.6 - 1.3) | 0.7 (0.4 - 1.2) | 1.0 (0.6 - 1.4) | 1.0 (0.6 - 1.5) | 0.7 (0.4 - 1.4) |
| **ST (h/day)** | **Before pandemic** | 0.6 (0.5 - 1.0) | 1.0 (0.5 - 2.0) | 2.3 (1.5 - 3.5) | 0.7 (0.5 - 1.0) | 1.0 (0.5 - 1.8) | 2.1 (1.5 - 3.1) |
|  | **During lockdown** | 1.1 (0.7 - 2.0) | 2.0 (1.0 - 3.4) | 5.0 (3.0 - 6.1) | 1.2 (1.0 - 2.0) | 2.0 (1.2 - 4.0) | 4.0 (3.0 - 6.0) |
|  | **Second wave** | 0.8 (0.5 - 1.1) | 1.3 (1.0 - 2.0) | 3.0 (2.0 - 4.0) | 1.0 (0.5 - 1.1) | 1.5 (1.0 - 2.0) | 3.0 (2.0 - 3.7) |
| **Sleep (h/night)** | **Before pandemic** | 10.0 (9.5 - 10.5) | 9.5 (9.0 - 10.0) | 8.0 (8.0 - 9.0) | 10.0 (9.1 - 10.3) | 9.5 (9.0 - 10.0) | 8.3 (8.0 - 9.0) |
|  | **During lockdown** | 10.0 (10.0 - 11.0) | 10.0 (9.0 - 10.0) | 9.0 (8.0 - 9.9) | 10.0 (9.5 - 10.5) | 10.0 (9.0 - 10.0) | 9.0 (8.0 - 10.0) |
|  | **Second wave** | 10.0 (9.5 - 10.3) | 9.0 (8.5 - 10.0) | 8.0 (7.5 - 8.6) | 10.0 (9.0 - 10.0) | 9.0 (9.0 - 10.0) | 8.0 (7.9 - 9.0) |

Abbreviations: PA: physical activity; sleep: sleep duration; ST: screen time; h: hours; y: years

Time points: before pandemic: before March 2020; during lockdown: between 16 March and 10 May 2020; second wave: between October 2020 and January 2021.

Data are median (P_25_-P_75_).

## Table S7. Unadjusted associations of number of recommendations met and adherence patterns during the second wave of the pandemic with well-being in early 2021 (*Corona Immunitas,* Switzerland, 2020-2021)

|  | **Excellent health** | | **Life satisfaction** | |
| --- | --- | --- | --- | --- |
|  | **OR (95% CI)** | **p-value** | **β (95% CI)** | **p-value** |
| **Number of recommendations met** |  |  |  |  |
| No recommendation met | Reference |  | Reference |  |
| One recommendation met | 1.37 (0.97 to 1.96) | 0.081 | 0.30 (0.08 to 0.51) | 0.008 |
| Two recommendations met | 1.83 (1.31 to 2.57) | <0.001 | 0.68 (0.48 to 0.89) | <0.001 |
| All three met | 2.20 (1.56 to 3.13) | <0.001 | 0.92 (0.71 to 1.14) | <0.001 |
| **Adherence patterns** |  |  |  |  |
| No recommendation met | Reference |  | Reference |  |
| PA only | 1.03 (0.60 to 1.75) | 0.901 | 0.22 (-0.11 to 0.54) | 0.194 |
| Sleep only | 1.38 (0.91 to 2.11) | 0.128 | 0.34 (0.08 to 0.6) | 0.010 |
| ST only | 1.50 (1.01 to 2.25) | 0.045 | 0.29 (0.04 to 0.54) | 0.023 |
| PA + Sleep | 1.88 (1.18 to 3.01) | 0.008 | 0.66 (0.36 to 0.95) | <0.001 |
| PA + ST | 1.61 (1.01 to 2.58) | 0.045 | 0.63 (0.33 to 0.93) | <0.001 |
| Sleep + ST | 1.86 (1.32 to 2.64) | <0.001 | 0.70 (0.49 to 0.92) | <0.001 |
| All three met | 2.20 (1.56 to 3.13) | <0.001 | 0.92 (0.71 to 1.14) | <0.001 |

Abbreviations: PA: physical activity; sleep: sleep duration; ST: screen time; β: regression coefficient; CI: Confidence interval; OR: odds ratio.

## References

1. West EA, Anker D, Amati R, Richard A, Wisniak A, Butty A, et al. Corona Immunitas: study protocol of a nationwide program of SARS-CoV-2 seroprevalence and seroepidemiologic studies in Switzerland. *Int J Public Health*. 2020;65:1529–48. doi:10.1007/s00038-020-01494-0.

2. Swiss Federal Statistical Office. *Demographic balance of the permanent resident population, by canton and commune*, 2010-2020. 2021. https://www.bfs.admin.ch/bfs/en/home/statistics/population/effectif-change/regional-distribution.assetdetail.18344222.html. Accessed 1 Oct 2021.

3. Ulyte A, Radtke T, Abela IA, Haile SR, Braun J, Jung R, et al. Seroprevalence and immunity of SARS-CoV-2 infection in children and adolescents in schools in Switzerland: design for a longitudinal, school-based prospective cohort study. *Int J Public Health*. 2020;65:1549–57.
